# Supplementary material for: Are carbon emissions trading and green financial instruments synergistic? -Comprehensive quantitative research based on content analysis
Source: PLoS One. 2024 Mar 7;19(3):e0298601. doi: 10.1371/journal.pone.0298601 (PMC10919601; doi:10.1371/journal.pone.0298601)
Supplement: S1 Data — (ZIP) [file pone.0298601.s001.zip › Data/地级市Z_F工作报告.zip!/╜¡╦╒/─╧═¿╩╨/└┤╫╘╣1⁄2╓┌║┼í╛┬φ┐╦╩2╛▌═°í┐_2011.docx]

各位代表：

　　现在，我代表市人民政府向大会作工作报告，请予审议，并请市政协委员和其他列席人员提出意见。

一、2010年工作回顾和“十一五”主要成就

　　过去一年，全市上下在省委、省政府和市委的正确领导下，齐心协力完成了市十三届人大三次会议确定的目标任务。地区生产总值3417.9亿元，按可比价计算增长13%；财政总收入713.4亿元、增长46.8%，其中，地方一般预算收入290.8亿元、增长46.1%；全社会固定资产投资2168.4亿元、增长20.3%；社会消费品零售总额1268.3亿元、增长18.7%；城镇居民人均可支配收入21825元、增长12.1%，农村居民人均纯收入9914元、增长14%；城镇登记失业率2.75%；完成节能减排目标任务。

　　（一）转型升级取得新进展。

　　新兴产业势头良好。海洋工程、新能源、新材料等七大新兴产业产值1615.8亿元、增长31.4%。海洋工程完工量389.4万综合吨、增长50.9%，产量占全国市场份额的1/3，成为国家新型工业化产业示范基地。风电并网发电装机容量64.2万千瓦、增长18%。光伏电池产量523兆瓦、增长62.9%。规模以上新兴产业企业总数突破1000家，在建超亿元项目135个。

　　传统产业扩量提质。船舶及配套产业产值966.7亿元、增长21.3%，规模以上造船完工量808万载重吨、增长42.5%，我市成为国家船舶高新技术产业化基地。纺织服装产业产值1694.3亿元、增长16.7%，电子信息、精细化工等产业产值增幅均超过25%。家纺、茧丝绸等成为省级特色产业基地。新增省级以上名牌产品70个，驰名、著名商标40件，家纺版权保护经验被世界知识产权组织确定为优秀典范。建筑业施工产值2700亿元、增长23.7%。

　　服务业发展速度加快。服务业增加值1246.9亿元、增长13.6%。规模以上服务业投资600.8亿元、增长36.1%。物流业、金融业增加值占服务业比重分别达到17.1%和11.3%。港口货物吞吐量1.5亿吨。旅游总收入230.2亿元、增长27.9%。市区高星级酒店建设取得突破性进展，新增6家，客房总数增长64%。服务外包营业额8.1亿元、增长70%。

　　集约发展、节能减排力度加大。13个省级以上开发区以全市4%的面积，创造了全市48%的生产总值、43%的财政总收入。50个工业集中区销售收入占全市的60.2%，提高3个百分点。连续19年保持耕地占补平衡。全面完成省下达淘汰落后产能任务。关闭、搬迁小化工企业50家，印染、钢丝绳行业污染整治初见成效。新建扩建污水处理厂32座，新增污水日处理能力20.2万吨。海安县在全省江北率先建成国家生态县，如皋等4个县（市）基本达到国家生态县考核标准，全市85%的乡镇建成国家级生态乡镇。

　　自主创新能力增强。高新技术产业产值2424.1亿元、增长31.8%，占规模以上工业的32.4%。中远船务研发中心成为我市首家国家级企业技术中心；新增省级工程技术研究中心121家；科技孵化器面积突破200万平方米，新增国家级科技孵化器3家，南通科技园建成开园；南京大学南通材料研究院等启动建设。建立2个国家级、8个省级产业技术创新战略联盟，实施产学研合作项目400个，专利授权量突破2万件、列全省第三。22项科技成果获省级以上科学技术奖。“江海英才”计划全面实施，全市投入人才专项资金2.2亿元，引进领军型人才185名，4人入选国家“千人计划”，22人入选省“双创”计划；新增院士工作站15家。

　　（二）江海开发呈现新气象。

　　大产业项目加快集聚。组织472个亿元以上项目集中开工，总投资超过2000亿元。大唐吕四港电厂、江苏LNG接收站、王子制纸一期等百亿级项目投产或竣工。沿江沿海前沿地区完成规模以上投资1071.5亿元、增长23.6%。

　　大港口开发加快实施。洋口港10万吨级北航道工程完工，管线桥、太阳岛二期等建成；吕四港区吕四作业区5万吨级码头投运，东灶港作业区2个2万吨级码头开工；通州湾（暂定名）建港条件研究全面启动。长江口-12.5米深水航道先导段贯通，新通海沙岸线整治工程海门段竣工，狼山港三期集装箱泊位、如皋港10万吨级通用泊位建成。全市新增5万吨级以上泊位5座。

　　大通道建设加快推进。20项重大交通基础设施项目进展顺利。崇启大桥完成总投资的66%。沪通铁路公路大桥开工准备工作基本就绪。宁启铁路南通段复线电气化改造工程开工，海洋铁路完成工程量的76.3%。江海高速建成通车，通洋高速、临海高等级公路、平海公路开工建设，干线通道沿海边布局的态势已经形成。兴东机场飞行区改扩建完成工程量的65%。连申线航道南通段整治工程开工，焦港船闸建成。

　　大园区承载功能加快提升。国家级开发区和12个省级开发区新落户10亿元以上重大项目26个。苏通科技产业园一期基础设施工程基本竣工，研发中心等7个配套项目开工。锡通科技产业园、上海市北高新（南通）科技城、海宝工业园、中国供销产业园、上海外高桥（启东）产业园、杨浦（海安）工业园等跨江合作园区进入实质性开发阶段；洋口港南通国际产业园开工。实施滩涂围垦项目11个，新增可用滩涂17.4万亩，沿海开发空间进一步拓展。

　　（三）改革开放迈出新步伐。

　　开放型经济加快转型升级。举办港洽会、世界船舶高层峰会和“龙腾江海·耀眼南通”等系列招商活动，新批协议外资49.8亿美元，注册外资实际到账29亿美元、增长44.6%。新批及增资超9000万美元项目16个，新批装备制造和新兴产业项目占比达到42%。进出口总额211亿美元、增长29.8%；机电、高新技术产品出口占比分别达到42.7%、19.1%。完成外经营业额12.5亿美元，继续保持全省领先；境外投资额突破3亿美元，为上年的7.4倍。外事、侨务、港澳事务、对台事务、口岸工作在扩大开放中发挥重要作用，新缔结国际友城2个。

　　民营经济发展质态加快提升。个体工商户总数、私营企业数和注册资本等指标保持全省第二。民营经济增加值占地区生产总值比重60%，提高2个百分点。新引进市外民资项目注册资本536亿元、增长55%。新增汽车及配件等百亿级特色产业板块4个。24家民营企业入围全国500强。新增以民企为主体的上市企业9家，总数22家，熔盛重工成为我国当年在香港上市的最大民营企业，中小板创业板上市企业数进入全国大中城市前十强。

　　各项重点改革任务加快实施。新一轮市县政府机构改革、乡镇机构改革任务基本完成。长江镇、吕四港镇被列为全省首批扩权强镇试点镇。南通市农村商业银行成立，华夏、民生银行南通分行开业，广东发展银行南通分行获准筹建，银行业金融机构总数达到32家。全省江北首家外资银行日本名古屋银行南通分行通过开业验收。省信用再担保公司在我市设立全省首家分公司。市级国有资产公司专业化经营加快，资本运作平台作用有效发挥。文化、医药卫生体制改革和事业单位全员聘用改革稳步推进。

　　（四）“三农”工作获得新成效。

　　现代农业发展进一步加速。规模以上“三资”开发农业投入增长24.2%，农业综合开发投入增长37.8%。新增高效农业39.7万亩、高效渔业11.3万亩，新增省级现代农业产业园区2家、农业龙头企业68家。江海台湾农民创业园启动建设。市区实施都市生态型农业项目46个。粮食连续7年增产，单产创历史新高。

　　农村基础设施和公共服务水平进一步提升。新建改造农村公路1350公里、农村公路桥梁和农用桥梁845座。新增城乡客运线路15条。江海堤顶硬质化改造262公里。完成第三批70万农村居民饮水安全工程建设任务。引江调水规划启动实施。农村河道提前实施新一轮疏浚。建成4个垃圾焚烧发电厂。新增造林面积17.3万亩。村级劳动保障服务平台实现全覆盖，60%的行政村建成“充分就业村”；新增农村劳动力转移5万人，总量列全省第一。

　　农村发展活力进一步增强。认真落实各项强农惠农政策，市、县财政“三农”投入37.4亿元、增长39%。“万顷良田试点工程”稳步推进，新增土地流转面积31万亩。农民专业合作组织入社率提高5.3个百分点，新增土地股份合作社239家，社区股份合作制改革面83%。村级集体经济快速发展。农村小额贷款公司“三农”贷款增长158%，农业政策性保险覆盖面进一步扩大。

　　（五）城市功能有了新提升。

　　城市建设统筹推进。修编新一轮城市总体规划，编制完成市区路网等4类28项专项规划。市区组织实施城建项目230个，完成投入136亿元；建成区面积125.2平方公里，增加31.4平方公里。各县（市）城市总体规划修编全面启动，城市框架加快拉开。11个省级重点中心镇联结城乡的纽带作用进一步发挥。

　　中心城市功能增强。新城区竣工和在建商务、金融等功能性项目37个，总面积238万平方米，核心区建设初具规模。老城区改造建设力度持续加大，环濠河商业中心改造步伐加快，寺街·名人文化区建设前期工作启动；文峰城市广场、圆融广场等一批城市综合体项目全面启动。开发区能达商务区总部大厦等项目开工。港闸区一批老镇改造和历史文化保护项目顺利推进，城市绿谷一期建成，华强城市乐园等一批旅游休闲、商务居住项目正式落户，北翼新城核心区建设加快实施。通州城东新区等四大功能片区框架全面拉开。实施市区“102030”交通畅通工程。已建和在建快速路总里程超过40公里，通沪大道高架建成通车，江海大道快速化改造等5个项目开工。城市组团间交通联接进一步强化，北城大桥、园林路、钟秀东路东延段等竣工，静海大桥、长江南路改造等开工。新购和更新公交车133辆，新辟公交线路20条。新增日供水能力40万吨、污水日处理能力7.5万吨。推进39个老小区环境整治，其中23个基本完成。新增绿地240公顷。市、区两级数字化城管平台建成运行，市容环境卫生面貌不断改善。

　　（六）民生保障得到新加强。全市财政民生投入168.5亿元、增长31.4%，占一般预算支出的54.5%。10件27项政府为民办实事项目全面完成（见附件一）。引江区域供水等12项超额完成年度目标，食品安全动态监测等15项完成年度目标。全市引江区域供水工程普及率73%，新增饮用长江水受益人口150万人，如皋市实现区域供水乡镇全覆盖。

　　就业和社会保障工作不断强化。全市新增城镇就业9.96万人，城镇失业人员再就业3.85万人。城镇职工五大保险覆盖率均稳定在97%以上，工伤保险实现市级统筹；城镇居民基本医疗保险覆盖率98%，新型农村合作医疗参合率99.5%；新型农村社会养老保险覆盖率93%，为108.7万农村老年居民发放基础养老金；城镇老年居民开始享受养老补贴。对城乡80岁以上老人普遍实施优待金制度，市区居家养老服务站全覆盖。企业退休人员养老金和城乡低保标准再次提高，市区被征地农民独立保障体系初步建立。完成脱贫攻坚阶段性任务。

　　住房保障力度加大。全市拆迁安置房新开工536.6万平方米、竣工交付391.5万平方米，均增长一倍以上，其中，市区竣工交付269.8万平方米、增长106.9%，安置拆迁户1.35万户；全市提供公共租赁住房1.1万套（间）、经济适用房4091套、廉租房465套。市区按照三年建成1万套的目标，启动建设保障性（限价）商品房。市区将符合条件外来务工人员纳入住房保障；5个"城中村"改造地块开工，3个片区危旧房改造项目基本竣工；普通商品住房用地出让353公顷、增长22%，新开工258万平方米、增长40.5%。

　　社会事业发展加快。制定实施南通市中长期教育改革和发展规划。完成校舍安全改造151.7万平方米。素质教育整体推进。我市被列为国家高职教育改革发展综合试验区。优化商贸高职、工贸技校等职教资源布局。南通大学被确定为博士学位建设单位。农村三级医疗卫生服务体系不断健全。市区基层医疗卫生服务机构全面实施国家基本药物制度。公益性文化设施基本实现镇村全覆盖。市图书馆新馆、凤凰国际书城、报业新闻传媒中心等文化设施开工。有线电视用户突破200万户，有线数字电视用户新增100万户。巩固全国文明城市创建成果，开展"讲文明、树新风、迎世博"主题活动，加强公民道德建设，社会文明程度进一步提高，城市公共文明指数测评列全省第二。全民健身体系进一步健全。我市成为全国人口和计划生育综合改革示范市。第六次人口普查登记任务完成。职工权益维护、妇女、儿童、青少年、残疾人、关心下一代、老区扶贫开发、见义勇为、法律援助、红十字和慈善等工作进一步加强。对口援建四川地震灾区三年任务两年完成。民族、宗教、地方志、统计、机关事务管理、气象、防震、档案、保密、新闻出版、哲学社会科学等工作不断加强。

　　（七）社会稳定取得新业绩。

　　积极创新社会管理体系，基层基础工作不断深化，社会稳定风险评估、社会平安信息发布等机制初步建立，我市成为全省唯一入选的全国社会管理创新综合试点城市，综治平安建设综合绩效列全省第一。乡镇（街道）政法综治工作中心全覆盖，专业社工服务、心理矫治中心等功能不断提升。万人发案率全省最低。“12345”政府服务热线、市长信箱、市长接待日、群众来信来访、网上与群众直接交流“五位一体”的民生诉求服务机制不断健全。建立政府网络发言人制度。“五五”普法任务全面完成，法治南通建设进一步加强。安全生产事故发生数和死亡人数连续9年双下降。食品药品监管和市场价格监管扎实有效。应急管理体系进一步健全。国防教育、民兵预备役、优抚安置、民防（人防）、国家安全、防邪反邪工作取得新成绩。

　　各位代表！过去一年，我们以推进政府机构改革为契机，进一步转变职能，着力提升工作创造力、行政执行力、政府公信力，不断加强自身建设。强化为民服务举措，建立民生诉求首问、首办负责制；强化重大项目会办服务，积极化解发展难题。加强调查研究，30多项调研成果转化为推进工作的举措；加大督办查办力度，133项重点工作全面完成。坚持依法行政，规范政府行政行为。推进政府信息公开，全面实施行政权力网上公开透明运行。自觉接受市人大及其常委会的法律监督、工作监督和市政协的民主监督，认真执行市人大及其常委会决议，支持市政协参政议政。对市人大“强化政策驱动，促进南通服务业又好又快发展”、“健全完善南通市食品安全检验检测体系工作”两个代表议案和市政协“加快我市特色园区建设”、“加强我市医疗卫生服务体系建设”两个建议案，专题交办、专人跟办、专项督办，促进了重点工作的落实。人大代表建议、政协委员提案办理任务全面完成。贯彻落实廉政准则，推进廉政教育，全面推行政府投资项目代建制，强化绩效审计，领导干部廉洁自律意识得到增强。

　　各位代表！“十一五”以来，面对复杂多变的外部环境，特别是面对国际金融危机冲击，全市上下以科学发展观为统领，紧紧围绕“全面达小康、建设新南通”的目标，加快经济转型升级，大力保障改善民生，取得了超出预期的发展成就。

　　这五年，综合实力大幅提升，城市位次显著前移。2010年，地区生产总值是“十五”末的2.3倍，在全国大中城市排名由“十五”末的第29位前移至第26位，列全国地级市第8位。地方一般预算收入是"十五"末的4倍，总量进入全国地级市十强、列全省第四。进出口总额突破200亿美元，列全国大中城市第25位。

　　这五年，江海开发取得历史性突破，南通昂首迈入桥港新时代。经过多年持之以恒努力，苏通大桥建成通车，崇启大桥即将贯通，沪通铁路公路大桥即将开工，洋口、吕四两大深水海港正式通航，结束了“有江无桥、有海无深水海港”的历史，综合交通枢纽门户城市格局加速形成。通州撤市设区，中心城市框架全面拉开，各县（市）城建设全面提速，城市化率提高到55%，大城市格局加速形成。苏通科技产业园等一批跨江联动、跨国合作园区启动建设，省级以上开发区、滨海产业园区承载功能显著提升，江海并进的大园区开发格局加速形成。各县（市）均成为全国综合实力百强县（市），崇川、通州、如皋、海门、启东地方一般预算收入均超过30亿元，南北区域协调发展格局加速形成。

　　这五年，全面小康目标提前实现，人民生活持续改善。2009年在全省江北率先总体达到省定全面小康标准。城镇居民人均可支配收入、农村居民人均纯收入连年保持两位数增长，2010年分别比“十五”末增长88.3%和80.2%；居民储蓄存款余额比“十五”末增长126%。形成“十大类二十二项”社会保障体系，城镇职工五大保险和新型农村合作医疗、新型农村社会养老保险基本全覆盖。2010年城乡低保标准分别比“十五”末提高67%、180%。全市财政民生投入550多亿元，为民兴办实事58件132项。

　　这五年，发展环境进一步优化，精神文明建设和社会建设管理不断强化。争创全省最佳办事环境成效明显。文化大市建设迈出大步，在全省江北率先以县域为单位基本实现教育现代化，公共卫生服务体系城乡全覆盖。大调解、大防控机制不断健全，公众安全感持续位居全省前列，荣获全国社会治安综合治理“长安杯”。全国文明城市、国家历史文化名城等“五城同创”目标圆满实现，城市美誉度进一步提升。大力推进思想道德体系和社会诚信体系建设，精神文明“南通现象”发扬光大，“包容会通、敢为人先”的新时期南通精神不断弘扬，全市上下进一步形成了政通人和、心齐气顺、风正劲足的良好氛围。

　　各位代表！五年的不懈拼搏，为“十二五”发展奠定了坚实基础，全市上下意气风发、斗志昂扬，南通正站在一个新的历史起点上，这必将进一步激励我们信心百倍、豪情满怀地开创南通更加美好的未来！

　　“十一五”发展所取得的成就，归功于省委、省政府和市委的正确领导，得益于历届领导班子打下的良好基础，离不开市人大、市政协的监督支持，凝聚着全市人民的智慧和汗水。在此，我谨代表市人民政府，向广大干部群众，向人大代表、政协委员和离退休老同志，向市各民主党派、工商联、无党派人士、各人民团体和各界人士，向人民解放军、武警驻通部队官兵、公安干警和部省驻通单位，向所有关心和支持南通发展的海内外朋友，致以崇高的敬意和衷心的感谢！

　　各位代表！南通的发展还存在不少亟待解决的问题，我们的工作还存在不少薄弱环节。经济结构中的深层次矛盾仍较突出，自主创新能力需要增强；城市功能和城镇化水平仍待提高，农村公共服务仍然不足；在城镇住房、拆迁安置、城市交通等方面还存在群众不满意的地方。少数政府工作人员存在形式主义、官僚主义和消极腐败现象。对此，我们一定高度重视，认真解决。

二、“十二五”时期的目标任务

　　各位代表！从今年开始，我们进入了本世纪第二个黄金发展的十年。十年看五年。根据《中共南通市委关于制定南通市国民经济和社会发展第十二个五年规划的建议》，市人民政府制定了《南通市国民经济和社会发展第十二个五年规划纲要（草案）》（以下简称《纲要（草案）》，提请大会审议。

　　“十二五”时期，我们将高举中国特色社会主义伟大旗帜，以邓小平理论和“三个代表”重要思想为指导，深入贯彻落实科学发展观，按照党的十七届五中全会、省委十一届九次全会和市委十届七次全会精神，紧紧围绕“加快现代化、再创新辉煌”的战略定位，全力抢抓两大国家战略规划深入实施的重大机遇，以科学发展为主题，以加快转变经济发展方式为主线，以改革开放和科技创新为强大动力，以保障和改善民生为根本目的，以全国社会管理创新综合试点城市为重要抓手，统筹做好改革发展稳定各项工作，促进经济长期平稳较快发展和社会和谐稳定。

　　按照“加快现代化、再创新辉煌”的战略定位，我们将在以下三个方面奋力拼搏：加快推进现代化建设，为2018年左右在全省江北率先基本实现现代化奠定具有决定性意义的基础；全力打造长三角北翼经济中心，促进沪苏通“小金三角”和沪甬通一体两翼格局加速形成；在全国、全省和长三角城市中持续赶超，争先进位。为此，《纲要（草案）》提出“六个更加”的新目标：

　　——经济更加强盛。经济发展主要指标增幅超过全省平均水平，到2015年，地区生产总值超过6000亿元，人均地区生产总值突破8万元。产业结构明显优化，发展质量和效益明显提升，服务业增加值占地区生产总值比重提高到41%。城市化和城市现代化水平明显提高，城市化率达到64%，城乡、区域发展更趋协调，成为江苏新的重要增长极。

　　——科教更加发达。创新能力显著增强，研发投入占地区生产总值比重力争达到2.5%，科技进步贡献率提高到60%；教育强市建设迈出坚实步伐，教育现代化水平进一步提高；人才强市战略取得明显成效，基本建成创新型城市。

　　--社会更加和谐。各项社会事业加快发展，城乡各类公共服务水平显著提升。法治南通、长安南通建设水平不断提高，民主法制建设进一步健全，社会公平正义得到进一步保障，公民文明素质进一步提高；社会管理创新、和谐社会建设走在全省和全国前列，社会大局持续稳定。

　　--文化更加繁荣。江海特色文化强市建设成效显著，文化事业和产业得到大力发展，公共文化服务体系基本建成。更高水平全国文明城市创建深入推进，新时期"南通精神"得到弘扬光大，精神文化生活更加丰富多彩，文化软实力大幅提升。

　　--生态更加优良。建成国家生态市，单位地区生产总值能耗和二氧化碳、主要污染物排放总量显著下降，森林覆盖率提高到21%，人民群众对生态和人居环境满意度持续提高。

　　--人民更加幸福。社会就业更加充分，城乡居民收入普遍较快增长，努力实现居民收入增长与经济发展同步，城镇居民人均可支配收入、农村居民人均纯收入分别达到4万元和1.9万元，力争五年实现倍增；社会保障体系日益完善，人民群众享有较高水平的教育、健康服务和公共文化产品，幸福感明显提高。

　　实现"十二五"发展目标，我们必须把握科学发展主题和转变发展方式主线，抓好产业现代化、城市现代化、人民生活现代化三大战略重点，强化科技创新、改革开放、社会管理创新、政治建设四大战略支撑。为此，《纲要（草案）》提出13项重点任务。完成好这些任务，需要我们突出创新引领，加快转型升级；突出沿海开发，放大发展优势；突出改革开放，激发体制活力；突出城乡一体，强化统筹发展；突出民生优先，促进社会和谐。

　　各位代表！我们坚信，到2015年，全市综合实力、自主创新能力、国际竞争力和可持续发展能力将显著增强，人民群众将普遍过上更加宽裕安康的生活，南通将在科学发展道路上创造又一次新的辉煌！

　　三、2011年主要任务

　　2011年是实施"十二五"规划的开局之年。做好今年工作，既要充分看到国内外经济环境的积极因素，进一步抢抓长三角一体化发展和江苏沿海开发两大国家战略叠加的黄金机遇，不断放大比较优势；又要深刻认识面临的新情况、新问题，始终保持清醒头脑，有效应对，趋利避害，奋力实现"十二五"良好开局。

　　今年全市经济社会发展的主要预期目标是：地区生产总值增长12%左右；地方一般预算收入增长15%以上；固定资产投资增长18%，社会消费品零售总额增长17%；外贸进出口总额增长10%以上，注册外资实际到账25亿美元；城镇居民人均可支配收入、农村居民人均纯收入均增长12%以上；居民消费价格涨幅控制在4%左右；城镇登记失业率控制在4%以内；全社会研发投入占地区生产总值比重力争达到2%；万元地区生产总值能耗降幅和主要污染物减排完成省下达任务。

　　实现上述目标，我们将按照市委十届七次全会和全市经济工作会议部署，坚持不懈调结构、抓创新、促转型，持之以恒重统筹、惠民生、促和谐，着力做好以下七个方面工作：

　　（一）加大产业结构调整力度。

　　加快发展战略性新兴产业。实施"新兴产业倍增计划"，抓好100个以上超亿元项目、100家优势企业、10个特色产业基地，新兴产业产值突破2000亿元。围绕打造国家级海洋工程产业基地，提升高端产品研发能力和总承包、专业化分包能力，海洋工程完工量超过450万综合吨。围绕打造国家绿色能源示范基地，加快建设"海上三峡"，风电并网发电装机容量超过90万千瓦；光伏电池及组件产能超过1300兆瓦。围绕打造长三角新材料研发转化生产基地，拉长功能性、专用型合成材料产业链。加快培育一批具有自主知识产权、自主品牌的生物技术药物，提升生物医药产业水平。着力突破数字化及系统集成技术，积极发展智能装备产业。大力开发推广高效节能装备及产品，壮大节能环保产业。

　　提高"千亿级"产业板块竞争力。推动信息化与工业化深度融合，加快推进船舶及重装备、能源及其装备制造两大产业"双倍增"，电子信息、石化两大产业"双突破"，纺织服装、轻工食品两大产业"双提升"，六大产业产值全部突破千亿元，新增百亿级工业企业1-2家、亿元以上工业企业100家。船舶产业重点向"三高一优"方向发展，加快打造"世界船谷"。能源及其装备制造重点发展高端、大型输变电和发电配套设备，加快打造千万千瓦级电力能源基地和全国重要的能源装备制造基地。电子信息重点做优光通信、新型电子元器件、集成电路测试封装等，加快打造技术先进的电子信息产业基地。石化产业重点向产业链两端延伸，强化新产品开发，加快打造全国重要的新兴石化产业基地。纺织服装以研发、生产、贸易一体化为方向，巩固提升"南通家纺"品牌，加快打造国内一流的家纺产业基地。轻工食品重点提升高档纸制品、醋片醋纤、电动工具等优势产品开发、品牌创建能力，加快打造华东地区具有较强影响力的轻工业基地。

　　推动现代服务业跨越发展。深入实施服务业发展三年行动计划，抓好168个市级服务业重点项目建设，服务业增加值占地区生产总值比重提高1个百分点。推进制造业企业主辅分离，生产性服务业增加值占服务业增加值比重提高1个百分点。大力发展服务临港产业、辐射长江中上游的港口物流，积极发展航空物流；培育、整合16个10亿元以上专业市场，壮大、提升2个百亿级大型专业市场。大力培育总部经济和会展品牌。壮大网络呼叫、动漫设计等特色产业，打造长三角北翼服务外包首位城市。加快发展现代商贸、社区服务、家政服务等生活性服务业。加强旅游大项目建设，旅游总收入力争达到280亿元。推动服务业集聚发展，40个市级以上服务业集聚区营业收入达到1200亿元。

　　大力推进自主创新。实施新一轮"高新技术产业双倍增"工程，高新技术产业产值超过3000亿元。加大科技投入，提升企业自主创新能力，培育更多自主知识产权和自主品牌，鼓励企业组建产业技术创新战略联盟，推动企业建设研发平台200家以上。实施产学研合作项目200个，新增科技孵化器50万平方米、入孵企业400家，引进1-2家重大研发机构。加快科技公共服务平台建设，推动创新资源共享。大力发展科技金融，支持和鼓励金融机构组建南通科技银行、科技创新担保公司、科技创业风险投资公司。深入实施"江海英才"计划，大力推进"百千万"人才引进和培养工程、"226"高层次人才培养工程，引进高层次创新创业领军人才和紧缺专业优秀人才2000名以上。建设富有竞争力的"人才特区"，解决好人才关注的居住、医疗和子女教育等问题。

　　坚持抓好节能减排和资源节约。加快淘汰落后产能，实施循环经济项目100个。推进工业园区生态化，开展低碳园区和企业创建试点。抓好100个节能降耗技术改造项目，新增污水日处理能力15万吨。严格执法和监管，落实重点企业、行业和区域节能减排责任。坚持最严格的耕地保护和节约用地制度，推进国家级节水型社会试点市建设，提高集约节约利用水平。

　　（二）推动沿海开发全面突破。

　　在基础设施建设上求突破。交通基础设施完成投资突破200亿元。推动沪通铁路公路大桥开工建设和崇启大桥尽快建成通车。宁启铁路南通段复线电气化改造完成总工程量的1/4以上，海洋铁路完成铺轨，宁启铁路通启段开工，海安至盐城铁路复线改造工程完成可研。兴东机场飞行区改扩建工程竣工。加快推进通洋高速、临海高等级公路建设和225等省道改扩建工程，建成平海公路东段。加强内河航道规划建设，抓好连申线航道南通段整治工程。做好崇海大桥、海启高速、城市轨道交通等项目前期工作。实施14个电力基础设施项目。推进洋口港15万吨级蓝沙洋北航道和5-15万吨级液体化工码头等建设。实施吕四港区挖入式港池和10万吨级进港航道、5万吨级港池码头等工程，建成东灶港作业区2个2万吨级通用码头。加快编制通州湾整体开发规划，启动腰沙促淤和通州三夹沙一期工程。提升沿江港口功能，有序推进长江北支、横港沙、如皋北汊等整治开发和保护。

　　在产业集聚发展上求突破。抓好总投资4658亿元的170个市级重点项目，完成投资超过760亿元。推动沿江船舶海工、精细化工等产业有序向沿海转移。强化国家级开发区和12个省级开发区大项目、大产业集聚功能。引导滨海产业园区完善规划，提升功能，吸引更多符合产业导向的项目落户。推进重点工业集中区创建示范、生态、特色园区。强势推动跨江联动、多方合作，加快苏通科技产业园一批总投资超亿美元重大项目建设，促进锡通科技产业园、海宝工业园、中国供销产业园、中国江海产业园、杨浦工业园、洋口港南通国际产业园等园区尽快形成项目集聚效应。

　　在推进统筹开发上求突破。按照全市江海联动开发功能布局和规划，强化市级统筹、共建共享的综合开发机制。推进省百万亩滩涂围垦综合开发试验区工程，实施4个区域59.4万亩滩涂围垦项目，力争完成10万亩。坚持生态开发、高效利用，加强资源的统一规划和管理。强化沿海开发融资平台建设，吸引大型央企、知名外企、民企参与沿海开发，引进市外民资400亿元以上。

　　（三）进一步深化改革扩大开放。

　　提升对外开放的层次和水平。实施"六千六百六十工程"，推动开放型经济转型升级。突出招引重特大项目，强化产业链招商、资源优势招商、环境品牌招商，引导外资更多投向沿海地区、新兴产业、现代服务业，打造日资、港资、台资集聚的开放型经济示范城市。创新招商方式，加大引园区、引业态、引技术和引才智力度，鼓励设立区域总部和研发、采购中心，吸引跨国金融、贸易等机构和创投、股权投资基金落户。突出提质增效，推动外贸发展方式转变。在船舶海工、机电、家纺等领域，培育一批高层次出口基地和大户，提高自主知识产权、自主品牌产品出口比重。突出跨国经营，拓展外经合作空间。支持有条件的企业到境外收购研发机构、营销网络、知名品牌及上市融资，建立资源开发及加工基地，积极承接国际总包工程和我国援外项目。进一步加强外事、侨务、港澳事务、对台事务和口岸开放工作。

　　加快民营经济转型升级。继续争创江苏民营经济第一大市，抓增量，调结构，全面提升发展质态。深入推进全民创业，打造"政策洼地"和"服务高地"，激发群众创业热情；完善财税、担保等扶持措施和"金融超市"等融资机制，优化创业环境和平台。大力推动集约发展，巩固提升家纺、电动工具等14个百亿级特色板块，新增2个百亿级特色板块。拓展专业施工领域，提高装备作业水平，建筑业施工产值突破3000亿元。坚持抓好"三名"工程，鼓励和支持有条件的民营企业通过多种形式实施整合重组，加快向规模化、品牌化、国际化方向发展。

　　加强重点领域改革。发展壮大各类金融机构，引进市外金融机构2-3家。推进农村金融体制改革。健全中小企业融资担保体系。加大企业上市工作推进力度，新增上市企业5家。完善土地、技术、人力资源等要素市场。健全国有企业经营业绩考核制度，确保国有资产保值增值。实施公共卫生与基层医疗卫生事业单位绩效工资，有序推进事业单位工资改革。

　　（四）统筹城乡发展，整体提升"民富村美风气好"的新农村建设水平。

　　加大城乡一体化发展力度。加快推进城乡规划、产业布局、基础设施、公共服务、劳动就业一体化。全面推进县（市）城市总体规划、乡镇总体规划修编。支持各地做大做强县（市）城，加强滨江临海新城、重点中心镇建设以及老集镇改造，建设县（市）城与重点中心镇之间的快捷通道，更好发挥城镇对城乡统筹发展的联结作用。稳妥推进"万顷良田试点工程"和农民集中居住区建设，积极推进户籍制度改革，促进工业向园区集中、人口向城镇集中、居住向社区集中、土地向适度规模经营集中。强化城乡生态环境建设和保护，狠抓生态林业重点工程，加强生态海洋建设；加大污水收集管网建设力度，生活污水集中处理率提高5个百分点；组织国家生态市创建攻坚，力争所有县（市）都通过国家生态县（市）考核验收。

　　放大高效农业规模优势。继续争创全省高效农业规模化第一市，新增高效农（渔）业33万亩以上，其中设施农（渔）业11万亩以上。加快农业产业集聚，打造百亿级特色产业板块。建设高标准农田18万亩。抓好江海台湾农民创业园建设，推进市级以上现代农业园区、基地、示范村创建，建成省级园区、省级特色基地8个以上。启动"农业产业化龙头企业和农民专业合作组织倍增工程"，加强农民经纪人队伍建设，提高农业市场化和组织化程度。大力发展外向型农业和品牌农业。加快构建产前、产中、产后一条龙的农业社会化服务保障体系。

　　优化农村发展环境。新建农村公路1200公里，新建改造农村公路桥梁和农用桥梁650座。加大引江区域供水推进力度，突破进村入户管网与干线管网衔接难点，引江区域供水工程普及率80%以上，新增饮用长江水受益人口68万人、农村饮水安全人口43万人。加大引江调水力度，建立农村河道轮浚机制，提高农村河道、道路、绿化、生活垃圾"四位一体"环境长效管理水平，农村生活垃圾无害化处置率100%。实施清洁家园工程，加强农业面源污染治理。强化农村教育、医疗、文化等公共服务，大力发展镇村公交。深入开展文明村镇、卫生村镇创建。加快发展村级集体经济。社区股份合作制改革面达到100%。开展农民资金互助合作试点，扩大农业政策性保险试点。

　　（五）强力推进中心城市建设。

　　大力增强城市集聚辐射功能。按照新一轮城市总体规划定位，拓展城市建成区规模，推动中心城市人气提升、商气兴旺。加快市域人口和外来人口集聚，提高市区城镇人口和经济总量占全市的比重。完善专项规划，高质量编制控制性详细规划，强化城市规划的前瞻性、操作性和控制力。开展城市综合交通枢纽规划研究。以总投资180亿元的107个重点基础设施项目为抓手，组织开展市区新一轮城建大会战。以推进总建筑面积超400万平方米、总投资超200亿元的28个城市综合体、商贸金融和文化教育医疗项目为重点，做大做强做靓新城区商务中心；以启动南大街步行化改造等工程为重点，做优做精做特老城区环濠河商业中心，加快崇川区为主体的城市核心区建设。以开发区能达商务区和苏通科技产业园功能配套为重点，加快南翼新城建设。以港闸区北大街沿线开发和上海市北高新（南通）科技城为重点，加快北翼新城建设。以拉开通州城区框架、提升要素集聚功能为重点，加快东部新城建设。继续抓好一批"城中村"、危旧房片区改造和老小区综合整治工程，加快推进观音山、唐闸、天生港等老街区改造。

　　大力优化城市交通。加快实施市区"102030"交通畅通工程，通过三年努力，基本实现主城区机动车辆10分钟上快速路、20分钟上高速、30分钟通勤。按照"一环一轴八射"快速路网布局规划，推进长江中路高架和东方大道、金通公路快捷化改造等12项工程，初步形成快速路系统。推进工农路北延、金通三大道等45项骨干道路工程以及28项支路建设和节点改造工程，不断完善路网体系。推进9个公共停车设施工程和15个居民小区停车设施建设，缓解部分公共区域、居民小区"停车难"。启动实施"公交优先20项"工程，新辟、优化公交线路38条，新购和更新公交车200辆，加快公交专用车道建设，提升出租车营运能力和管理水平，开工建设汽车客运枢纽东站。强化交通管理，建设智能交通，扩大单行系统。

　　大力提升城市形象和品位。突出挖掘中心城市文化底蕴，着力打造历史与现代交相辉映的江海魅力之城，彰显"中国近代第一城"新风采。新建一批城市精品建筑、精品小区，提高城区主要节点、标志性建筑的建设品位，整治提升商贸集聚区、主要通道沿线城市景观。加快推进寺街·名人文化区、西寺、伶工学社、南通·1895、天生·古港风情区、民俗文化博览园、中华慈善博物馆、南公园改造等一批历史文化保护、建设项目，华强城市乐园、城市绿谷二期、能达文化公园、"南通之眼"等一批文化、旅游休闲项目。以创建5A级景区为目标，进一步提升濠河、狼山景区绿化美化水平。推进通吕运河绿廊、星湖大道景观带等20个绿化项目，市区新增绿地200公顷以上。健全城市综合管理长效机制，强化数字化、精细化管理。加强市容环境整治和小区物业管理，确保通过国家卫生城市和国家环保模范城市复检。实施城市水系规划和市区"清水工程"三年行动计划，完成32条段市区河道整治。实施蓝天工程，空气质量优良天数比例保持在90%以上。市区启动城郊结合部"四位一体"环境长效管理。

　　（六）更大力度保障和改善民生。为民办好10件40项实事（见附件二），一般预算支出用于民生的比重提高0.5个百分点。

　　增加城乡居民收入。突出抓好高校毕业生、城镇就业困难人员、农村转移劳动力等重点群体就业，南通籍高校毕业生就业率95%以上，有就业愿望和能力的城乡登记失业人员就业率95%以上，80%的行政村建成"充分就业村"。推进职工工资集体协商，保障工资正常增长和及时支付，提高最低工资标准。营造更为宽松的政策环境，大力支持自主创业，让更多群众拥有经营性、财产性收入。强化扶贫和老区开发，巩固脱贫攻坚成果。完善社会救助机制，对生活困难群众继续实施价格上涨动态补贴。

　　提升城乡社会保障水平。扩大新型农村社会养老保险覆盖面。提高企业退休人员基本养老金和城镇老年居民养老补贴标准，建立市区城镇居民养老保险和职工自费补充医保制度。新型农村合作医疗人均筹资标准提高到250元，其中政府补助标准提高到200元、增长66.7%，县乡两级政策范围内住院费用补偿比例提高到65%以上；城镇职工和居民医保制度规定范围内医药费用报销比例分别达到80%和60%。推进各类社会保险市级统筹。健全城乡低保、农村五保户、被征地农民基本生活保障标准自然增长机制。抓好"菜篮子"工程，市域建设蔬菜保供基地5万亩，基本建成南通农副产品物流中心。加大市场价格预警和监管力度，坚决查处串通涨价、哄抬物价等行为，保持物价基本稳定。

　　坚决遏制房价过快上涨。落实各级政府住房保障主体责任。强化保障，遏制房价。加大住房保障专项资金筹集力度，优先保证用地供应，市区新开工拆迁安置房350万平方米、竣工交付200万平方米，安置拆迁户1万户；市区加快1万套保障性（限价）商品房建设，开工不少于5000套；市区建设公共租赁住房不少于4200套（间）；对符合申请廉租住房、经济适用房条件对象实现"应保尽保"，市区为符合条件的外来务工人员提供经济适用房。加大供应，遏制房价。合理确定新建住房价格控制目标，切实增加住房有效供给，市区普通商品住房用地出让不少于350公顷，新开工不少于300万平方米；提高中低价位、中小套型商品住房比重。加强监管，遏制房价。规范房地产市场秩序，对已出让土地超过两年没有开工建设的及时收回土地使用权；严格落实差别化的住房税收、信贷等政策，合理引导住房需求，有效遏制投机投资性购房，促进房地产业平稳健康发展。

　　（七）切实加强社会建设和管理创新。

　　加快发展社会事业。落实南通市中长期教育改革和发展规划，促进基础教育优质均衡发展，实施学前教育提升工程，推进中小学布局优化调整和校舍安全工程。抓好国家级高职教育改革发展综合试验区建设。加快实施新区高中、商贸高职新校区建设和体臣卫校迁建等项目。加强终身教育、特殊教育，保障困难群体和外来务工人员子女上学。大力发展公益性文化事业，完善镇、村（社区）公共文化服务网点。积极培育文化产业，引导文化消费，扶持发展文化创意、地方特色文化等产业。市区加快建设图书馆新馆、凤凰国际书城、报业新闻传媒中心、工青妇活动中心、文艺之家等文化设施，启动国际文化交流中心项目前期工作。完成县（市）有线电视数字化整体转换，乡镇广播电视服务站规范化建设比例超过80%。进一步弘扬精神文明"南通现象"和新时期"南通精神"，切实加强青少年思想道德建设和城乡志愿者服务工作，深入开展群众性精神文明创建活动，确保蝉联全国文明城市称号。加快推进医药卫生体制改革，全面实施国家基本药物制度，健全基层医疗卫生机构补偿机制；政府在每个乡镇办好一所卫生院，落实基本公共卫生及相关重大公共卫生服务项目；加强重大传染病防控工作；加快公立医院改革试点，优化优质医疗资源布局。深入开展全民健身运动。加强人口和计生服务，提高优生优育水平。应对人口老龄化加速趋势，加快养老服务体系建设。积极发展妇女、儿童、青少年、残疾人、关心下一代、见义勇为、法律援助、红十字和慈善等事业。推进民族、宗教、气象、防震减灾等事业协调发展。扎实做好地方志、哲学社会科学、统计、机关事务管理、档案、保密等工作。全力做好对口支援新疆、青海工作。

　　创新社会管理方式。以全国社会管理创新综合试点为契机，构建党政主导、部门负责、社会协同、公众参与的社会管理新格局，争创全国首批社会管理创新示范城市。推进社会管理"六大创新"和"十大体系"建设，实施社会管理创新26个重点项目。坚持一体化谋划，实施人本化管理，完善长效化机制，加快由控制型管理向服务型管理转变。强化源头管理，全面推行社会稳定风险评估制度，完善社会矛盾纠纷排查化解机制，加强特殊人群和外来人口服务管理。强化基础管理，大力推进"强基工程"，充分发挥基层社区组织、政法综治组织以及工青妇等群团组织、行业协会和商会等社团组织在社会管理中的重要作用。强化动态管理，创新发展社会矛盾纠纷大调解机制，完善"12345"政府服务热线等"五位一体"民生诉求服务机制，健全政府网络发言人制度，建成社会管理信息化平台。巩固"长安南通"成果，实施科技防范入户工程，提升社会治安综合治理水平，严密防范、依法打击各种违法犯罪活动，加强国家安全、防邪反邪等工作，健全公共突发事件应急管理机制，强化安全生产责任制和责任追究制。加强食品药品监管，保障人民群众饮食和用药安全。深入推进法治南通、诚信南通建设，启动"六五"普法。支持国防和军队建设，推进全国双拥模范城"五连冠"创建活动，抓好全民国防教育、征兵、民兵预备役、民防（人防）等工作。

　　各位代表！面对新形势、新任务，我们将紧紧围绕建设服务型政府、责任政府、法治政府和廉洁政府，进一步加强自身建设，不断提高工作创造力、行政执行力、政府公信力。进一步提升行政服务效能。巩固扩大政府机构改革成果，强化社会管理和公共服务。深入推进机关作风建设，以群众满意为第一标准，健全服务发展、服务民生的制度机制，严格落实首问、首办负责制，抓落实，讲效率，办实事。优化财政支出结构和方式，增强公共服务能力，提高公共服务水平。进一步提高依法行政水平。规范政府行政行为，完善行政执法程序，依法细化、量化自由裁量权。完善公众参与、专家论证、合法性审查和监督反馈等重大决策机制。提升行政权力网上公开透明运行水平，做好政府信息公开和新闻发布工作，提高行政透明度。严格执行政府规范性文件报备与审查制度。自觉接受人大及其常委会的法律监督、工作监督和政协的民主监督，高度重视办好人大代表议案、政协建议案和人大代表建议、政协委员提案。认真听取各民主党派、工商联、无党派人士和各人民团体的意见，主动接受社会各方面监督。进一步加强勤政廉政建设。保持奋发有为的精神状态，真抓实干，埋头苦干，敢于负责，勇挑重担。强化调查研究和跟踪督查，狠抓重要决策、重点工作、重大项目推进落实。强化绩效管理，严格行政问责，加大行政监察审计和责任追究力度。大力倡导艰苦奋斗，从严控制公费出国出境，加强公务用车管理，切实降低行政成本。全面落实党风廉政建设责任制，扎实推进政府系统党员干部思想道德体系和社会诚信体系建设，严肃查处违法违纪行为，努力保持公务员队伍为民、务实、清廉的良好形象。

　　各位代表！"十二五"发展的新征程已经开启，"加快现代化、再创新辉煌"的奋进号角已经吹响！让我们紧密团结在以胡锦涛同志为总书记的党中央周围，在省委、省政府和市委坚强领导下，万众一心，开拓创新，奋力拼搏，乘势而上，以优异成绩迎接建党90周年，为把南通的明天建设得更加美好而努力奋斗！
